# Co-Combustion of Sewage Sludge and Coal Slime: Kinetic Mechanisms and Slagging Characteristics Affected by Coal Slime Diversity

**DOI:** 10.3390/ma19214614

**Published:** 2026-10-30

**Authors:** Yanlin Wang, Jiayi Liu, Zhen Yuan, Jingkuan Li

**Affiliations:** School of Electric Power, Civil Engineering and Architecture, Shanxi University, Taiyuan 030006, China

**Keywords:** sewage sludge, coal slime diversity, co-combustion, kinetics, slagging

## Abstract

**Highlights:**

**What are the main findings?**

- *R_s_* and *R_z_* are reliable indices for predicting co-combustion slagging.
- Ash content in CS dictates co-combustion kinetics and slagging behavior.
- Slagging can be avoided with >40% CS and temperatures below 900 °C.

**What are the implications of the main findings?**

- This work provides a kinetic basis for efficient co-combustion of sewage sludge and coal slime, supporting the resource recovery of the two solid wastes.
- It offers practical guidance to suppress slagging by rationally setting the coal slime blending ratio and combustion temperature.
- The validated reliable slagging prediction indices help evaluate fuel blends and ensure safe operation of co-combustion systems.

**Abstract:**

Co-combustion enables the resource recovery of sewage sludge (SS) and coal slime (CS). This study examined how CS variability affects SS–CS combustion kinetics and slagging. SS and CS exhibited multistep thermal decomposition kinetics. The activation energy for SS combustion followed an “N-shaped” distribution with respect to the conversion rate, whereas the activation energy for the combustion of CS decreased gradually with increasing conversion rate and was positively correlated with ash content. Blending with CS reduced the apparent activation energy, notably by 20.0 kJ/mol at a 50% ratio, accompanied by a shift in the kinetic mechanism from *f*(*α*) = (1 − *α*) to *f*(*α*) = (1 − *α*)^1.3^ during fixed-carbon combustion. SS had a much higher slagging tendency than CS, consistent with the predicted results for the evaluation indices except *Fe*/*Ca*. The incorporation of CS markedly improved the ash-fusion characteristics of the blends. The sulfur slagging index and comprehensive ash index were reliable indices for predicting co-combustion slagging. Effective mitigation of slagging was achievable when the CS proportion exceeded 40% and the combustion temperature remained below 900 °C. These findings inform CS type selection, optimization of blending ratios and combustion temperatures, and slagging-risk assessment, providing an experimental basis for parameter selection and stable SS–CS co-combustion operation.

## 1. Introduction

The development of urbanization and the acceleration of industrialization have led to the generation of more organic solid wastes. Meanwhile, with the concept of a “zero-waste city”, the rationalized disposal of these organic solid wastes has gradually attracted attention. Municipal sewage sludge (SS) and coal slime (CS) are typical representatives of these organic solid wastes. SS is produced during sewage treatment, and the amount of SS generated in China currently exceeds 60 million tons/year (at 80% moisture content) [1]. A variety of hazardous components, including organic pollutants, pathogens, and heavy metals, are present in SS [2]. Without appropriate treatment, these substances pose risks to both the ecological environment and human health. CS is a by-product of the coal washing process. As the largest consumer of coal, China has the highest CS production in the world. Unfortunately, a large amount of CS is currently being disposed of haphazardly, resulting in land waste and environmental pollution. Therefore, finding efficient methods for the disposal of SS and CS is crucial in the current context.

SS and CS contain a large number of organic components, which have a certain calorific value (12–16 MJ/kg) after drying [3], and their disposal by incineration to recover the heat generated in the process has attracted extensive attention. In fact, incineration after drying has become a mainstream technical route for SS disposal. However, the energy consumed by drying before combustion affects the net energy recovery from SS and CS utilization. Drying requires heat to warm the material and evaporate moisture, and its energy demand depends on the amount of water removed and the drying efficiency. For SS, thermal drying also requires electricity for auxiliary equipment, while the heat supply configuration affects system energy efficiency [4]. In addition, wet CS is highly viscous, and its rheological behavior depends on moisture content and temperature, complicating transport and feeding. Previous research has shown that, within a certain moisture-content range, increasing the moisture content of CS reduces its viscosity and improves its flowability. However, the additional moisture also increases the heat required for subsequent drying or evaporation in the furnace [5]. Practical applications therefore require a balance between drying energy consumption and transport requirements, with coordinated selection of the dewatering degree, drying heat supply, and transport and feeding methods.

In addition, due to the characteristics of SS, such as high ash content, low calorific value, and high N and S contents, individual combustion is prone to instability and excessive emissions. Co-combustion of SS and CS provides a solution to this problem, i.e., the relatively high calorific value and high fixed-carbon content of CS compensate for the problems associated with the combustion of SS alone. Compared with SS, SS-CS blends have higher calorific values. They are expected to be processed in circulating fluidized bed boilers, which have wide fuel adaptability. In this process, the heat generated from combustion can be used to generate electricity. At the same time, the pollutants produced can be removed by the complete flue gas cleaning system of the circulating fluidized bed boiler.

At present, co-combustion, which leverages the complementary properties of different fuels, has been widely studied, mainly focusing on SS-coal, SS-biomass, and CS-biomass [6,7,8,9]. Ma et al. investigated the co-combustion behavior of SS and coal using a thermogravimetric analyzer and found that SS significantly improved the ignition and combustion performance of coal [10]. Dai et al. found that the combustion kinetics of fixed carbon in bituminous coal were improved by SS addition, and the apparent activation energy was also reduced [11]. Ni et al. found that both the ignition and combustion performance were improved during the co-combustion of SS and straw [12]. Wang et al. investigated the co-combustion of sewage sludge and wheat straw and found that the blending ratio affected the combustion characteristics and apparent activation energy of the blends. Within the range examined, the lowest average apparent activation energy was obtained at a wheat straw blending ratio of 50% [13]. Accordingly, multi-fuel co-combustion has significant advantages in improving combustion characteristics. More importantly, the co-combustion of fuel mixtures does not simply equal the sum of their separate combustion processes; instead, interactions between the fuels may take place, thereby influencing the combustion kinetics. It has been reported that the interactions during the co-combustion of SS-gangue and SS-biomass can achieve in-situ inhibition of pollutants, including NO, SO_2_ and heavy metals [14]. These studies support the feasibility of co-combusting SS with other solid fuels and indicate that co-combustion behavior depends on feedstock composition, blending ratio, and interactions between fuels.

In addition to combustion characteristics and pollutant emission behavior, ash slagging behavior is also a key factor in determining whether solid-waste co-combustion technology can be applied to coal-fired boilers. The slagging behavior mainly depends on the mineral composition of the fuel, and differences in mineral composition among various fuels may induce interactions between inorganic phases during co-combustion, thereby affecting ash slagging behavior. Qi et al. investigated the co-combustion of Zhundong coal and SS and found that Si, Al, and P in SS participated in the transformation of sodium-containing minerals and affected the occurrence of sodium in ash. Mineral reactions and physical dilution alleviated ash sintering under specific conditions [15]. Yu et al. combined experiments with thermodynamic analysis to investigate ash melting and high-temperature flow behavior during the co-combustion of different sewage sludges and coal. Their results indicated that feedstock ash composition and blending ratio affected ash melting and flow behavior [16]. Deng et al. found that adding CS increased the ash melting point of Zhundong coal [17]. However, excessive Fe in the ash lowered the ash melting point by destabilizing the silicates [18]. In addition, alkali and alkaline earth metals are also important factors contributing to slagging problems during combustion [19]. The high content of Fe, alkali metals, and alkaline earth metals in SS poses a potential risk of slagging during co-combustion.

Differences in the composition and properties of CS influence its co-combustion with SS. Previous work compared the effects of CS type and pretreatment method on combustion characteristics and interactions [20] and investigated the associated kinetic behavior through the catalytic effects of SS mineral components and the combustion of fuel blends [21,22]. Microscopic observations of ash have also provided preliminary evidence that CS mitigates slagging during SS combustion [22]. Building on these findings, further comparisons are needed to clarify differences in the combustion kinetics of CS samples with different ash characteristics and in the melting and slagging behavior of their blended ash with SS. Whether different slagging indices reflect the observed slagging behavior of these fuels and their blended ash also requires evaluation against experimental results.

To address these questions, this study compared the evolution of activation energy and the stage-specific kinetic mechanisms during the individual combustion of XCS, TCS, and QCS, and used SS–TCS as a case study to examine the effect of blending ratio on co-combustion kinetics. The study focused on comparing the deformation and softening temperatures of SS–XCS, SS–TCS, and SS–QCS blended ash at different blending ratios. Six slagging indices were calculated from ash composition, and their applicability to the investigated systems was evaluated using macroscopic and microscopic observations of the ash. Building on previous research, this study provides comparative evidence of differences in CS reactivity and blended-ash slagging risk to inform CS selection and blending-ratio adjustment.

## 2. Materials and Methods

### 2.1. Materials

In this work, SS was collected from a wastewater treatment plant in Taiyuan City (Shanxi Province, China). The CS samples, denoted as XCS, TCS, and QCS, were obtained from three coal washing plants in the same city. After collection, all samples were dried, mechanically crushed, sieved, and dried again to achieve a particle size below 74 µm. Proximate and ultimate analyses of SS and CS are given in Table 1. SS and CS were then blended in different mass proportions (8:2, 6:4, 5:5, 4:6, and 2:8), forming mixtures named 8SS2CS, 6SS4CS, 5SS5CS, 4SS6CS, and 2SS8CS, respectively. The inorganic composition of both materials was determined using X-ray fluorescence spectroscopy, with the detailed data presented in Table 2.

Table 1 shows that SS had a volatile matter content of 47.44% and a fixed-carbon content of only 3.94%. Compared with SS, all three CS samples had lower volatile matter contents and higher fixed-carbon contents, indicating clear differences in the composition of their combustible fractions. The ash contents of the three CS samples increased in the order XCS < TCS < QCS, whereas their net calorific values followed the reverse order. The net calorific values of XCS and TCS were 26.15 and 15.19 MJ/kg, respectively, both exceeding that of SS (10.84 MJ/kg), whereas that of QCS was only 8.18 MJ/kg. Thus, the effects of CS on the energy characteristics of the blends depend on the CS type, and CS selection should consider combustible fractions, ash content, and calorific value together. These differences also provide a basis for comparing the combustion kinetics of different CS samples.

Table 2 shows clear differences in inorganic composition between SS and the three CS samples. Among the reported inorganic components, the Fe_2_O_3_ and CaO contents of SS were 43.6% and 10.6%, respectively, both higher than those of all three CS samples. In contrast, SiO_2_ and Al_2_O_3_ dominated the inorganic composition of the CS samples, with combined contents of 66.6–88.6%. QCS had the highest combined SiO_2_ and Al_2_O_3_ content, whereas TCS had the highest Fe_2_O_3_ content among the three CS samples. Blending therefore changes both the composition of the combustible fractions and the inorganic composition of the blended ash, which may affect ash melting and slagging behavior. These compositional differences provide a basis for subsequent analyses using ash fusion temperatures, slagging indices, and ash morphology.

### 2.2. Weightless Behavior

An STA449F3 thermogravimetric analyzer (NETZSCH, Selb, Germany) was employed to investigate the weight loss characteristics during combustion. Each test used roughly 10 mg of sample, which was heated from room temperature to 900 °C in air flowing at 100 mL/min. Three heating rates (5, 10, and 15 K/min) were applied. To ensure reliability, each condition was repeated three times, and the observed measurement deviation remained below 1%.

### 2.3. Combustion Kinetics

For the combustion reaction, the integral kinetic equation can be described as Equation (1) [23,24].

(1) $$G(\alpha )=\int_{0}^{\alpha }\frac{d\alpha }{f\left(\alpha \right)}=\frac{A}{\beta }\int_{T_{0}}^{T}\exp\left(-\frac{E_{a}}{RT}\right)dT$$

where *f*(*α*) is the mechanism function; *α* is the degree of conversion, %; *A* is the pre-exponential factor, min^−1^; *β* is the heating rate, K/min; *E_a_* is the apparent activation energy, kJ/mol; *T* is the reaction temperature, K; and *R* is the gas constant, *R* = 8.31 J/(mol·K).

(1) The FWO and KAS methods

The core of combustion kinetics lies in determining the three kinetic parameters that describe the combustion process: the pre-exponential factor *A*, the mechanism function *f*(*α*), and the apparent activation energy *E_a_*. Common approaches to non-isothermal kinetic analysis include single-heating-rate model-fitting methods and multiple-heating-rate isoconversional methods. The former require fewer experimental data, but the resulting kinetic parameters are sensitive to the assumed reaction model. In contrast, isoconversional methods estimate the apparent activation energy without assuming a specific reaction model, thereby reducing the influence of model selection. They also allow the variation in *E_a_* with conversion to be examined, providing a basis for analyzing multistep combustion processes. Their limitations include the need for experimental data at several heating rates, which increases the experimental workload, and the requirement that reaction states at the same conversion be comparable across heating rates. Furthermore, activation energies obtained using the FWO and KAS methods alone cannot uniquely determine the reaction model or pre-exponential factor, so further analysis using approaches such as the master-curve method is required. In this study, the isoconversional method was used to determine the apparent activation energy, *E_a_*, assuming that *E_a_* and the pre-exponential factor *A* corresponding to the same conversion are identical across different heating rates. The representative approaches mainly include the Flynn–Wall–Ozawa (FWO) and Kissinger–Akahira–Sunose (KAS) methods [25,26,27,28]. The general equations of the FWO and KAS methods are shown in Equations (2) and (3), respectively.

(2) $$\ln\beta =\ln(\frac{AE_{a}}{RG(\alpha )})-5.331-1.052\frac{E_{a}}{RT}$$

(3) $$\ln(\frac{\beta }{T^{2}})=\ln(\frac{AR}{E_{a}G(\alpha )})-\frac{E_{a}}{RT}$$

In the FWO method, a linear fit between ln(*β*) and 1/*T* was carried out under varying heating rates for a fixed conversion degree. The corresponding *E_a_* was then calculated from the slope of this fit. For the KAS method, the same conversion degree was used, but ln(*β*/*T*^2^) was regressed against 1/*T*, and the resulting slope gave the *E_a_*.

(2) The master curve method

Although relatively dependable *E_a_* estimates can be obtained from the FWO and KAS methods, neither approach provides information about *f*(*α*) or *A*. Therefore, the present study employed the master curve method [26], which is based on the FWO and KAS methods, to evaluate *f*(*α*) and *A*.

Let *u* = *E_a_*/*RT*, then Equation (4) can be obtained from Equation (1).

(4) $$G(\alpha )=\frac{A}{\beta }\int_{T_{0}}^{T}\exp\left(-\frac{E_{a}}{RT}\right)dT\approx \int_{0}^{T}\exp\left(-\frac{E_{a}}{RT}\right)dT=\frac{AE_{a}}{\beta R}\int_{\infty }^{u}\left(\frac{e^{-u}}{u^{2}}\right)du=\frac{AE_{a}}{\beta R}P(u)$$

The master curve method typically uses *α* = 0.5 as the reference value, and Equation (5) is derived from Equation (4).

(5) $$G(0.5)=\frac{AE}{\beta R}P(u_{0.5})$$

where *u*_0.5_ denotes the value of *u* at *α* = 0.5, Equation (6) can be obtained by dividing Equation (4) by Equation (5).

(6) $$\frac{G(\alpha )}{G(0.5)}=\frac{P(u)}{P(u_{0.5})}$$

The experimental master curves were obtained under different heating rates by plotting *P*(*u*)/*P*(*u*_0.5_) against *α*. The theoretical master curves, expressed as *G*(*α*)/*G*(0.5) versus *α*, were generated by substituting the mechanism-specific *G*(*α*) functions (Table 3) into Equation (6). The most probable *f*(*α*) corresponding to the experimental data was identified by evaluating the consistency between the experimental and theoretical curves. Once *f*(*α*) was known, the pre-exponential factor *A* was obtained using the previously calculated *E_a_*.

### 2.4. Methods for Studying Slagging Characteristics

The ash fusion temperature is a commonly used indicator for evaluating the slagging characteristics. In this study, an HKHR-4C intelligent ash fusion tester (Hebi Hengke Instrument and Meter Co., Ltd., Hebi, China) was employed to determine the ash fusion temperatures of blends with different mixing ratios, including the deformation temperature (*DT*) and softening temperature (*ST*). During the test, the furnace temperature was increased under programmed control, and a CCD imaging system recorded changes in ash-sample morphology during heating. *DT* and *ST* were determined from these morphological changes. In addition, ash samples of the co-blends at various temperatures were obtained using a muffle furnace. The inorganic composition of the co-combusted ash was analyzed using an X-ray fluorescence (XRF) spectrometer, based on which the base-to-acid ratio (*B*/*A*), silica–alumina ratio (*Si*/*Al*), silica ratio (*G*), iron–calcium ratio (*Fe*/*Ca*), sulfur slagging index (*R_s_*), and comprehensive ash index (*R_z_*) were calculated to evaluate the slagging tendency of the ash. The evaluation criteria for slagging characteristics based on ash fusion temperature and ash composition are presented in the Table A1 in Appendix A.

## 3. Results

### 3.1. Combustion Kinetics

**Figure A1:**
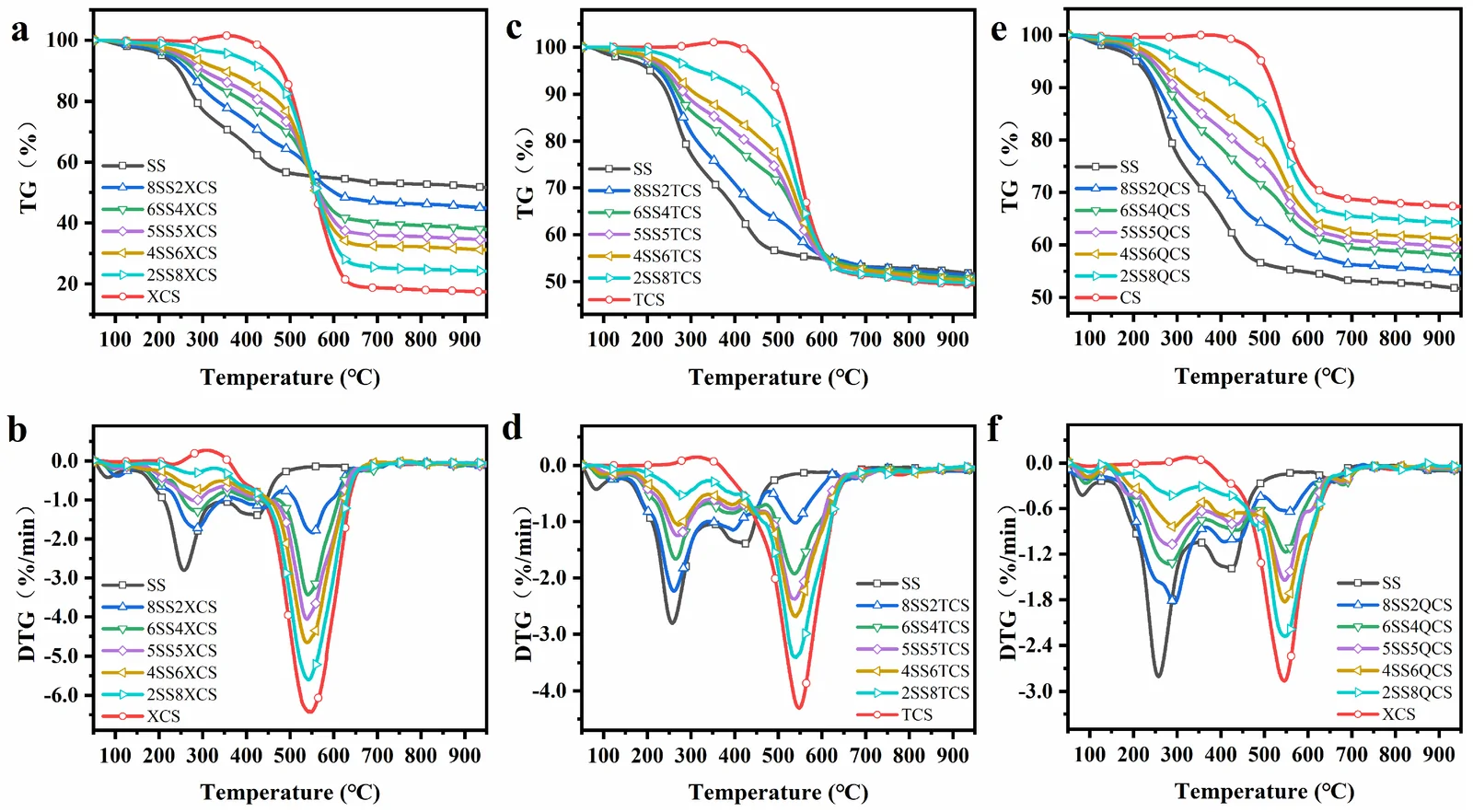
TG and DTG curves during SS–CS co-combustion: (**a**) TG and (**b**) DTG for SS–XCS; (**c**) TG and (**d**) DTG for SS–TCS; (**e**) TG and (**f**) DTG for SS–QCS.

The study of combustion kinetics helps reveal the intrinsic mechanisms of combustion, thereby providing theoretical guidance for the design of combustion devices in practical applications [29,30]. The combustion kinetics analysis was based on raw weight loss curve data obtained directly from thermogravimetric experiments (Figure A1 in Appendix A). The experiments were conducted in an air atmosphere at heating rates of 5, 10, and 15 K/min. Previous studies have reported the combustion characteristics of the samples (ignition temperature, combustion completion temperature, comprehensive combustion index, etc.) [20], these will not be repeated here. Instead, this paper focuses on the kinetic mechanisms of the combustion process, including the evolution of activation energy, the determination of mechanism functions, and the calculation of pre-exponential factors.

#### 3.1.1. Effect of Heating Rate on Thermogravimetric Behavior

**Figure 1:**
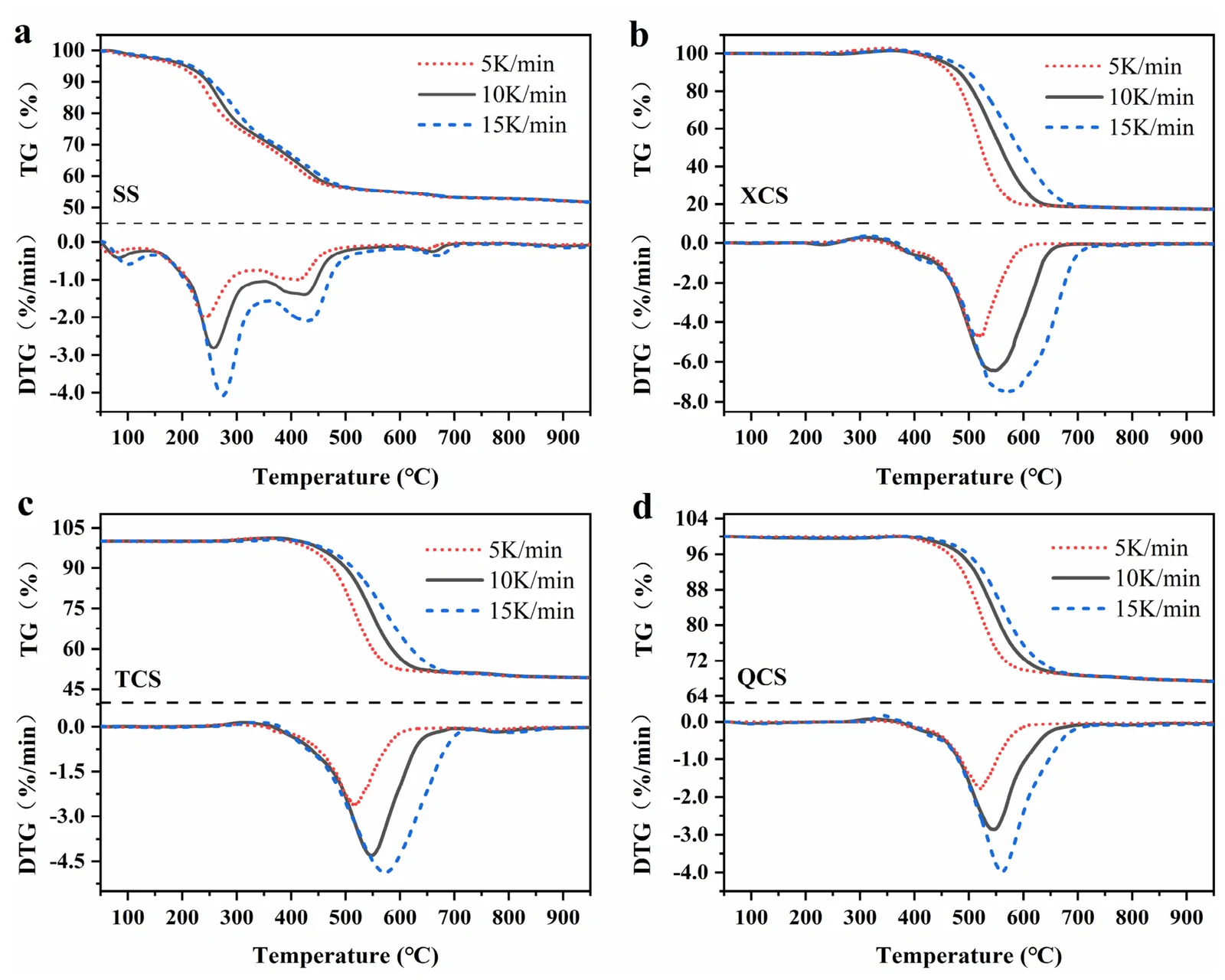
Effect of heating rate on the TG and DTG curves of (**a**) SS, (**b**) XCS, (**c**) TCS, and (**d**) QCS.

The TG and DTG results of SS, XCS, TCS, and QCS samples at different heating rates during combustion are shown in Figure 1. As shown in the figure, the weight loss behavior of all samples was generally similar at different heating rates, and the weight loss rate was not affected by the heating rate. However, both the TG and DTG curves of the samples shifted toward higher temperatures as the heating rate increases. This phenomenon is mainly attributed to the “thermal lag” effect [31,32]. During rapid heating, a large temperature gradient develops between the particle surface and the interior, increasing the difference between the actual reaction temperature and the measured temperature. This variation is reflected in the TG curve as a shift in the reaction process towards the higher temperature region. In addition, as the heating rate increased, the reaction temperature rose, and the weight loss rate of the samples increased accordingly. Moreover, an increase in the heating rate leads to a higher reaction temperature, resulting in a higher weight loss rate for the samples.

#### 3.1.2. Combustion Kinetics of SS and CS

**Figure A2:**
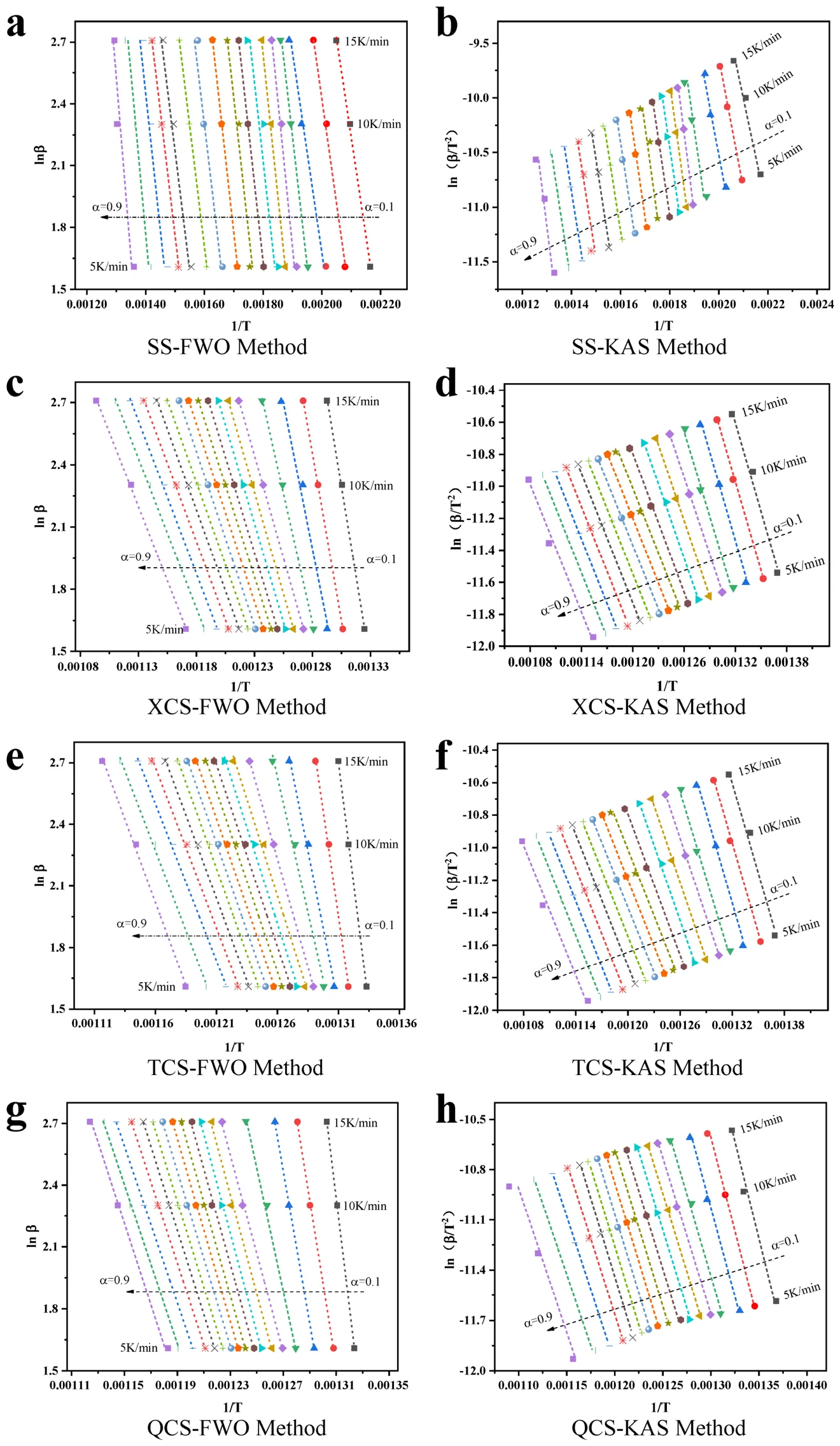
Linear fitting results obtained using the FWO and KAS methods: (**a**) SS–FWO, (**b**) SS–KAS, (**c**) XCS–FWO, (**d**) XCS–KAS, (**e**) TCS–FWO, (**f**) TCS–KAS, (**g**) QCS–FWO, and (**h**) QCS–KAS.

The Figure A2 in Appendix A shows the linear fitting results of 1/*T* versus ln*β* (or ln*β*/*T*^2^) obtained from the weight loss behavior during the individual combustion of SS, XCS, TCS, and QCS. Within the *α* = 0.10–0.90 conversion range, 1/*T* exhibited high linearity with ln*β* (or ln*β*/*T*^2^) (*R*^2^ > 0.95), verifying the applicability of the FWO and KAS methods for calculating the combustion activation energies of SS/CS.

**Figure 2:**
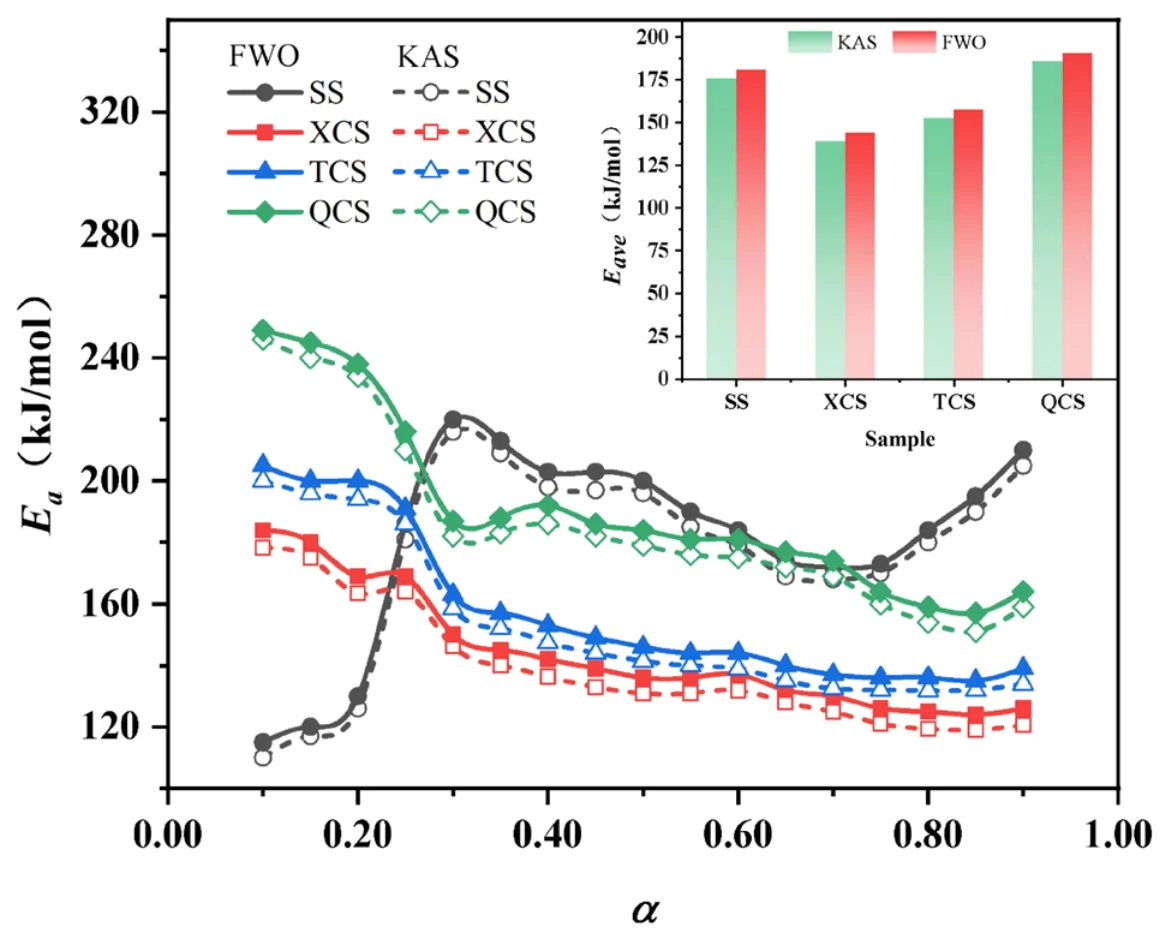
Evolution of activation energy with increasing conversion during SS/CS combustion.

The combustion processes of SS and CS involve complex chemical reactions during which numerous intermediate products are generated, each corresponding to the occurrence of an elementary reaction. Therefore, the activation energies obtained from the calculations in this study represent the apparent activation energies of the overall combustion reaction. The value of the activation energy during the combustion process is influenced by factors such as the concentration of activated molecules, diffusion mechanisms, and impurity concentrations [33]. Figure 2 shows the variation in *E_a_* with *α* during the combustion process of SS and CS.

As shown in the figure, the relative deviation of the *E_a_* values obtained by the FWO and KAS methods is within 5%, which mutually verifies the reliability of the two methods for calculating *E_a_* [13]. The *E_a_* during the combustion process of SS exhibited significant fluctuations with the variation in *α*, showing an “N-shaped” distribution, indicating that the combustion of SS can be classified as a multistep kinetic process that cannot be described by a single kinetic mechanism. When *α* ranged from 0.10 to 0.20, the *E_a_* remained at a relatively low level (approximately 125 kJ/mol). At this stage, the combustion reaction can occur by overcoming a lower energy barrier, which corresponds to the lower *T_i_* of SS mentioned earlier. However, when the conversion rate exceeded 0.20, the activation energy rose sharply to over 220.0 kJ/mol. Within the *α* range of 0.20–0.75, a gradual decline in *E_a_* was observed. This stage predominantly corresponds to the decomposition and combustion of heavy volatiles present in SS. The observed *E_a_* trend suggests that a relatively high energy barrier must be overcome during this period. As combustion proceeds, several factors, including heat accumulation, the development of a porous structure, and the catalytic influence of mineral components, contribute to the reduction in *E_a_* across the *α* = 0.20–0.75 interval [34]. When *α* > 0.75, the *E_a_* increased again, which is mainly attributed to the endothermic reactions and decomposition of minerals in SS, while the increase in ash content also results in a rise in *E_a_*.

The variation trend of *E_a_* during the combustion process of CS differed significantly from that of SS. Compared with SS, the *E_a_* of CS decreased gradually as *α* increased. At the initial stage of combustion, CS exhibited low oxidation activity and needed to overcome a higher energy barrier to initiate the combustion reaction. As the combustion process progressed, the heat released during the early stages accumulated continuously, while the porous structure of the char promoted gas diffusion, leading to a gradual decrease in *E_a_*. When *α* = 0.30, the activation energies of XCS, TCS, and QCS were 150.0 kJ/mol (FWO method), 163.1 kJ/mol (FWO method), and 186.9 kJ/mol (FWO method), respectively. When *α* > 0.30, the combustion of CS was primarily governed by fixed carbon, during which the variation in *E_a_* remained relatively stable, indicating that the combustion mechanism of the fixed carbon in CS is relatively simple. By comparing the *E_a_* variation trends across different types of CS, it can be concluded that ash content significantly influences *E_a_* during combustion. During the combustion process, the *E_a_* of QCS remained consistently higher than those of TCS and XCS, while XCS exhibited the lowest *E_a_* (123.9–150.0 kJ/mol, FWO method). This result indicates that the *E_a_* of CS is positively correlated with its ash content.

Among SS, XCS, TCS, and QCS, XCS exhibited the lowest average *E_a_*, with the values obtained by the FWO and KAS methods being 139.0 kJ/mol and 144.1 kJ/mol, respectively. The relatively low ash content (17.87%) is considered the main reason for this result. The ash content of QCS was as high as 69.58%, and consequently, it exhibited the highest average *E_a_*, with the values obtained by the FWO and KAS methods being 185.7 kJ/mol and 190.6 kJ/mol, respectively. The average *E_a_* of SS and TCS was 176.0 kJ/mol (KAS method) and 180.7 kJ/mol (FWO method) and 152.7 kJ/mol (KAS method) and 157.6 kJ/mol (FWO method), respectively.

**Figure 3:**
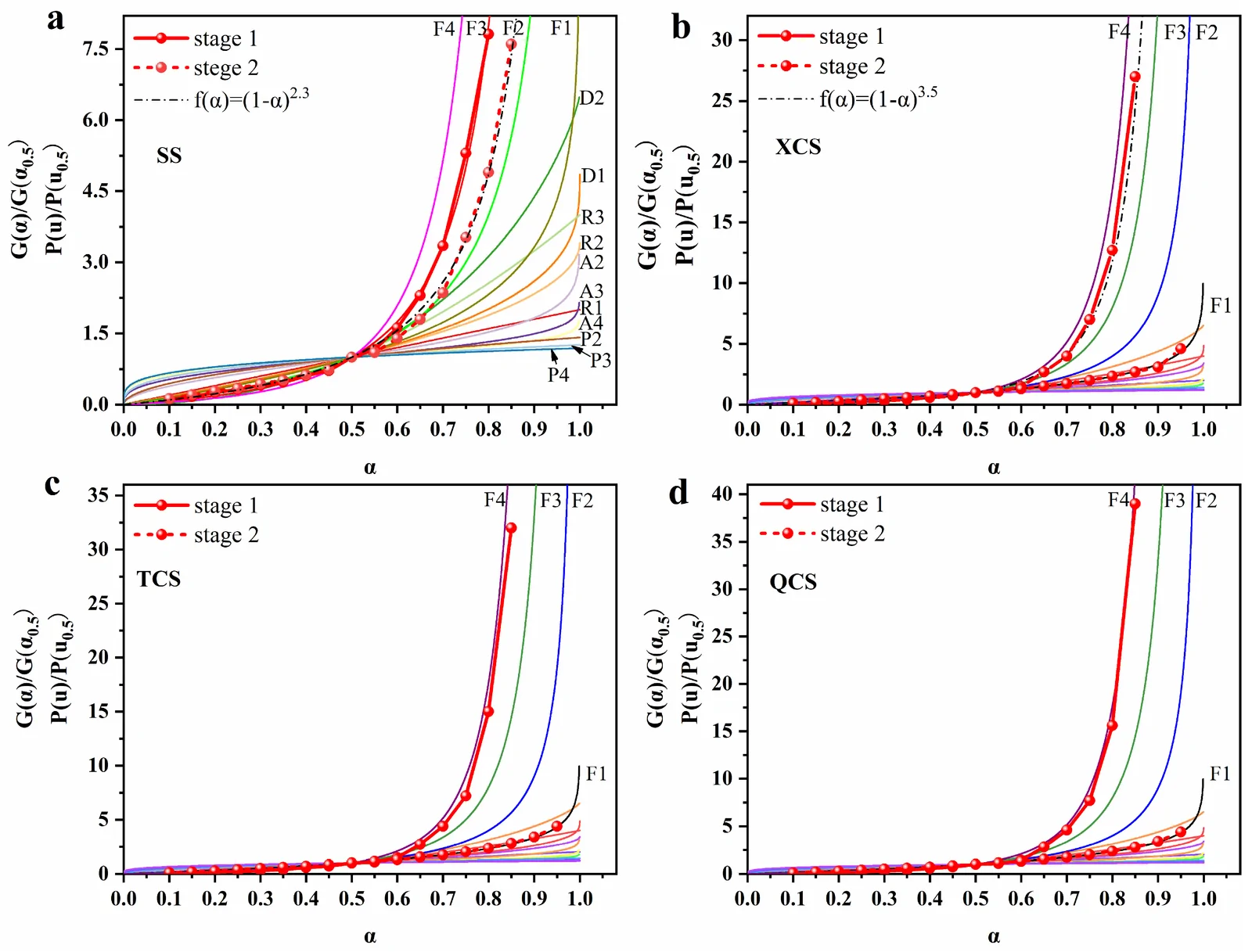
Comparison of theoretical and experimental master curves for (**a**) SS, (**b**) XCS, (**c**) TCS, and (**d**) QCS. The reaction models corresponding to codes F1–F4 are defined in Table 3.

Since the *E_a_* of SS and CS varied significantly with *α*, their combustion processes cannot be described by a single mechanistic function. Based on the trend of *E_a_*, the combustion process was divided into two stages: stage 1 and stage 2. For SS, Stages 1 and 2 correspond to *α* = 0.10–0.20 and 0.30–0.80, respectively; for CS, they correspond to *α* = 0.10–0.25 and 0.30–0.90, respectively. Within each stage, the difference between the maximum and minimum *E_a_* was less than 20% of the mean value. According to the recommendation of ICTAC (International Confederation for Thermal Analysis and Calorimetry) [24], the combustion reaction within each stage can therefore be considered to follow a single-step kinetic mechanism. Based on the obtained *E_a_* values, this study constructed theoretical master curves of *G*(*α*)/*G*(0.5) as a function of *α* using several commonly employed reaction models listed in Table 3, and compared them with the experimental master curves of *P*(*α*)/*P*(0.5) as a function of *α*. When the experimental and theoretical master curves coincide, the combustion kinetics can be considered to follow the *f*(*α*) corresponding to the theoretical master curve. The comparison results for SS, XCS, TCS, and QCS are shown in Figure 3. The combustion mechanisms of all four samples in the different stages followed the reaction-order model. For SS (Figure 3a), the combustion mechanism functions in stages 1 and 2 were *f*(*α*) = (1 − *α*)^3^ and *f*(*α*) = (1 − *α*)^2.3^, respectively. For the three types of CS (Figure 3b–d), the mechanism functions in stage 1 were *f*(*α*) = (1 − *α*)^3.5^, *f*(*α*) = (1 − *α*)^4^, and *f*(*α*) = (1 − *α*)^4^, respectively, while the combustion processes in stage 2 all followed the first-order reaction model, that is, *f*(*α*) = (1 − *α*).

Combining the n-th-order reaction model with Equation (4) yields the kinetic equations for SS and CS, as shown in Equation (7).

(7) $$\begin{array}{l} G(\alpha )=\frac{AE}{\beta R}P(u)=\frac{{\left(1-\alpha \right)}^{1-n}-1}{n-1},n\neq 1\\ G(\alpha )=\frac{AE}{\beta R}P(u)=-\ln(1-\alpha ),n=1 \end{array}$$

A linear regression of [(1 − *α*)^1−*n*^]/(*n* − 1) or −ln(1 − *α*) against *EP*(*u*)/*βR* was performed, and the pre-exponential factor *A* was determined from the slope. The fitting results at a heating rate of 10 K/min are presented in Table 4.

#### 3.1.3. Co-Combustion Kinetics of SS-CS

**Figure 4:**
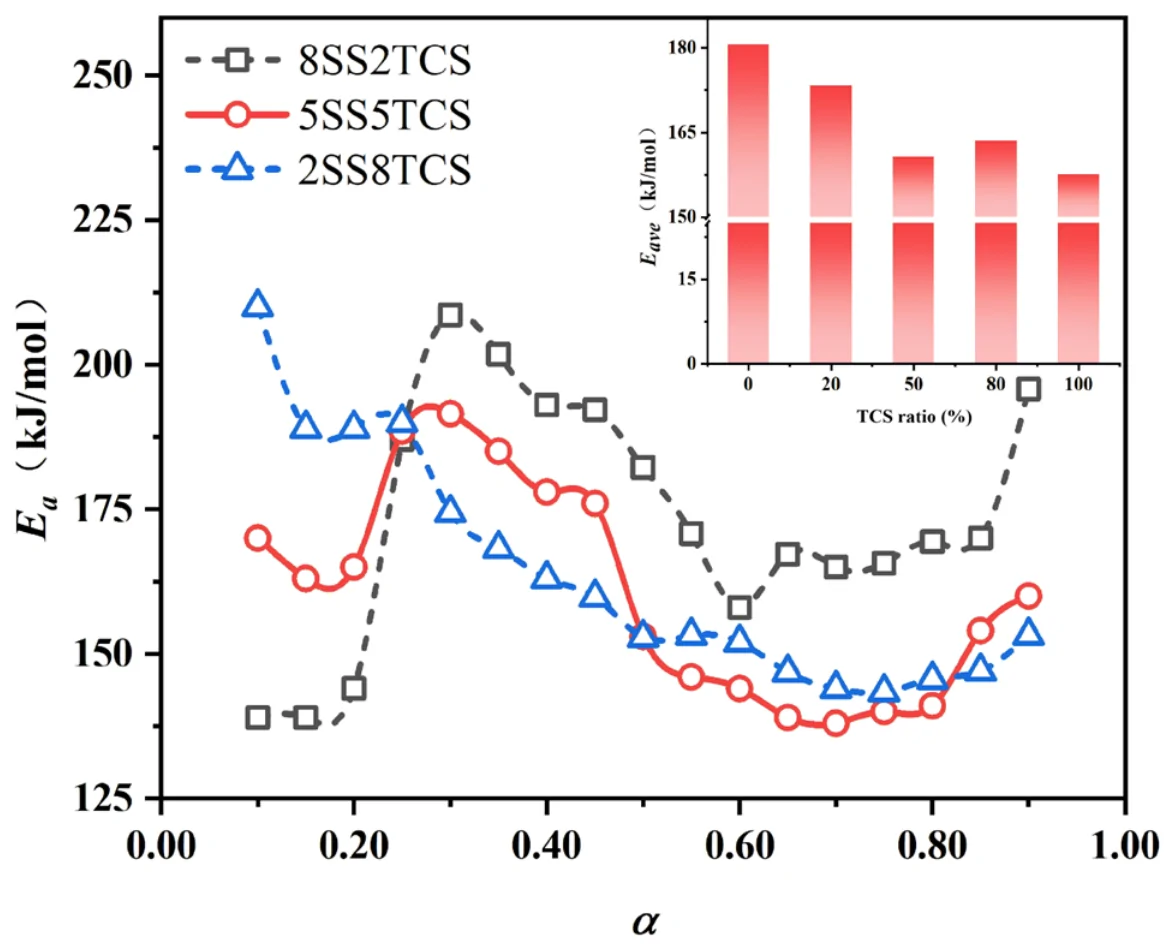
Variation in activation energy with conversion rate during combustion of SS-TCS.

TCS was selected for the co-combustion kinetic analysis based on its major fuel properties and practical utilization characteristics. Its ash, volatile matter, and fixed-carbon contents were 51.00%, 13.04%, and 35.58%, respectively, and its net calorific value was 15.19 MJ/kg (Table 1). Based on practical experience with CS utilization, these properties are close to those of comparable CS commonly used in engineering applications, making TCS relatively representative among the three CS samples selected in this study. SS–TCS was therefore used as a case study to further examine the effect of blending ratio on co-combustion kinetics. The FWO and master-curve methods were used for the analysis. Figure 4 shows the variation in *E_a_* with α for SS–TCS blends at different blending ratios.

As shown in Figure 4, the variation in *E_a_* during the combustion of the blends was more complex than that of the individual components, indicating that the co-combustion process involved more complex reaction mechanisms. Among these, the *E_a_* variations for 8SS2TCS and 5SS5TCS resembled the “N”-shaped profile of SS, whereas that of 2SS8TCS mirrored TCS, showing a gradual decrease with *α*. For *α* < 0.50, the *E_a_* of 5SS5TCS lay between those of 8SS2TCS and 2SS8TCS. When *α* was between 0.50 and 0.80, its *E_a_* was the lowest among the three samples. This stage corresponded predominantly to fixed-carbon combustion. Together with previous research on the influence of SS mineral components on CS combustion kinetics [21], this observation suggests that mineral components in SS may promote fixed-carbon combustion through catalytic effects. In terms of the overall average apparent activation energy, the addition of TCS reduced the *E_a_* of the co-combustion process. The extent of reduction exhibited a nonlinear relationship with the TCS proportion.

Among the three blending ratios examined, the lowest overall average apparent activation energy was obtained at a TCS proportion of 50%. The *E_a_* decreased by 20.0 kJ/mol relative to SS combustion alone, from 180.7 to 160.7 kJ/mol. To further evaluate this change, the average apparent activation energies for the individual combustion of SS and TCS were linearly weighted according to their mass fractions. At a TCS proportion of 50%, the resulting reference value was 169.15 kJ/mol, whereas the value calculated for the blend was 160.7 kJ/mol, which was 8.45 kJ/mol lower. This result is consistent with the interpretation of catalytic promotion during co-combustion and provides supporting evidence for the proposed role of SS mineral components in promoting fixed-carbon combustion.

As discussed above, the combustion of blends exhibited complex multistep kinetic behavior when *α* < 0.50. This behavior can be attributed to the increased diversity of volatiles introduced by TCS, which led to more intricate and variable reaction mechanisms. However, once the blends transitioned into the fixed-carbon combustion phase, the activation energy exhibited relatively little variation, implying that a single reaction mechanism governed the fixed-carbon combustion in these mixtures. Based on the average *E_a_* of the blends over the conversion range of 0.50 < *α* < 0.85, the mechanism function *f*(*α*) and pre-exponential factor *A* for this stage were determined using the master-curve method, and the results are presented in Table 5. The 5SS5TCS blend exhibited the lowest *E_a_* (144.4 kJ/mol) during the fixed-carbon combustion stage. Furthermore, compared with the combustion of individual components, a 20% SS addition had no effect on the combustion mechanism of TCS fixed-carbon. In contrast, higher SS additions of 50% and 80% shifted the mechanism function from *f*(*α*) = (1−*α*) to *f*(*α*) = (1−*α*)^1.3^ and *f*(*α*) = (1−*α*)^1.4^, respectively. Although most of the combustible constituents in SS have already been consumed before the fixed-carbon combustion stage during co-combustion, the combustion mechanism at this stage was still influenced by SS. This observation indicates that certain components in SS participated in the fixed-carbon combustion reaction of TCS [35]. The kinetic parameters obtained in this section and their variation with blending ratio characterize the SS–TCS system examined here.

### 3.2. Slagging Characteristics

#### 3.2.1. Ash Fusion Temperature

**Figure 5:**
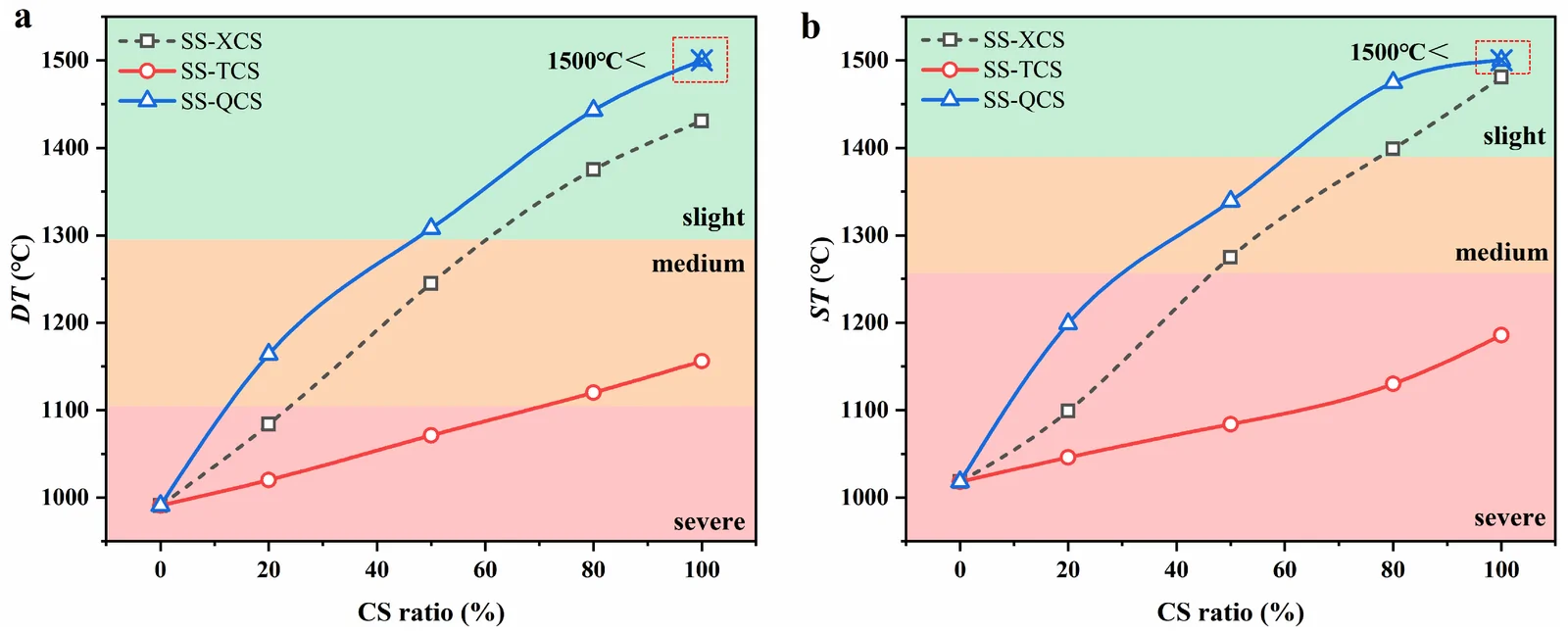
Ash fusion temperatures of SS–CS blends at different CS ratios: (**a**) deformation temperature (*DT*) and (**b**) softening temperature (*ST*).

The ash fusion temperature is a key parameter for evaluating slagging characteristics. In this study, the slagging tendency was assessed based on the deformation temperature (*DT*) and the softening temperature (*ST*). The *DT* and *ST* of the three blends at different blending ratios are shown in Figure 5.

Among SS and CS, SS exhibited the lowest *DT* and *ST*, at 991 °C and 1018 °C, respectively. The alkali metals, alkaline earth metals, and Fe in SS are likely responsible for its poor slagging characteristics [36]. Among the three types of CS, TCS showed the lowest *DT* and *ST*, at 1020 °C and 1064 °C, respectively, whereas the *DT* and *ST* of QCS both exceeded 1500 °C. The high SiO_2_ content of QCS ash, reaching 59.1%, increases the thermal stability of its crystalline structure [37]. During co-combustion of SS and CS, the incorporation of CS improved the fuel’s slagging characteristics, and both the *DT* and *ST* of the blends increased progressively with increasing CS proportion. Using *DT* and *ST* as evaluation indices, SS shows a severe slagging tendency, whereas both XCS and QCS are unlikely to slag. Compared to *DT*, the *ST* evaluation indicator provides a more stringent assessment of slagging tendency, resulting in a considerably broader composition range being identified as severely slagging than that determined using *DT*.

#### 3.2.2. Ash Composition

**Figure 6:**
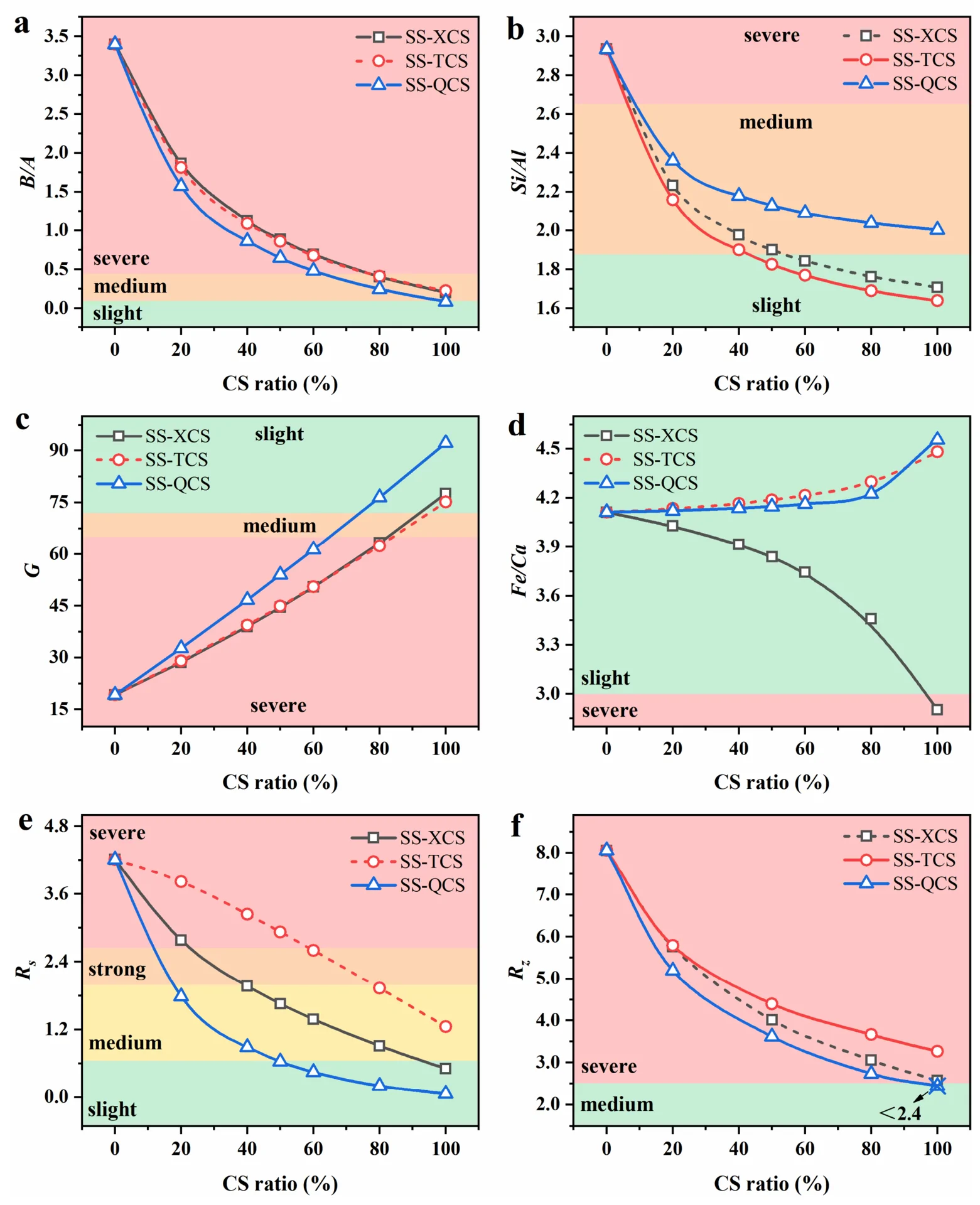
Slagging indices of SS–CS blends at different CS ratios: (**a**) base-to-acid ratio (*B*/*A*), (**b**) silica–alumina ratio (*Si*/*Al*), (**c**) silica ratio (*G*), (**d**) iron–calcium ratio (*Fe*/*Ca*), (**e**) sulfur slagging index (*R_s_*), and (**f**) comprehensive ash index (*R_z_*).

The slagging and fouling characteristics of fuels are related not only to the ash fusion temperature but also to the chemical composition of the ash. To more accurately investigate the slagging characteristics during the co-combustion of sewage sludge and coal, the chemical composition of the ash samples was analyzed, and various slagging indices were subsequently calculated. The effects of CS proportion on the slagging indices (*B/A*, *Si/Al*, *G*, *Fe/Ca*, *R_s_*, and *R_z_*) of the three blends SS-XCS, SS-TCS, and SS-QCS are shown in Figure 6a–f.

Except for *Fe/Ca*, all the other evaluation indices show that SS exhibited a severe slagging tendency. This is because SS contains large amounts of basic oxides such as CaO and Fe_2_O_3_, whereas the contents of acidic oxides such as SiO_2_ and Al_2_O_3_ are relatively low. Interactions among inorganic components in ash can affect ash melting and sintering. Slagging risk should therefore be evaluated in relation to the specific ash composition and mineral transformations [15]. For the CS samples, the slagging tendencies indicated by different evaluation indices varied, but all fall within the slight or moderate range, and the slagging risk was considerably lower than that of SS.

According to the *B/A* and *G* indices, the slagging tendencies of the three CS samples follow the order XCS ≈ TCS > QCS. For QCS, the *B/A* value was 0.086, corresponding to a slagging tendency classified as “low,” while the *G* value was 92, indicating a “slight” slagging tendency. However, according to the *Si/Al* index, QCS exhibited the most pronounced slagging tendency among the three CS samples, with a *Si/Al* value of 2.00, corresponding to a slagging tendency classified as “moderate”. Because the *B/A* and *G* indices fully account for the effects of Fe_2_O_3_ and CaO on slagging tendency, their results differ markedly from those of the *Si/Al* index, which considers only the silica–alumina components.

The *Fe/Ca* index, which assesses slagging tendency based solely on the Fe_2_O_3_/CaO ratio, yields results that diverge significantly from those of the other indices. It predicts that all samples except XCS (*Fe/Ca* = 2.9) were classified as non-slagging.

The *R_s_* index incorporates the effect of sulfur content on top of acidic and basic oxides. According to the *R_s_* results, QCS and XCS exhibited slight slagging tendencies (*R_s_* = 0.1 and 0.5, respectively), whereas TCS shows a moderate slagging tendency (*R_s_* = 1.2). Although the *R_z_* index demonstrates a trend consistent with *R_s_* in predicting the slagging tendency of the blends, its more stringent criterion classifies all blends except QCS as having a severe slagging tendency.

For the SS-CS blends, all indices except *Fe/Ca* consistently indicate a decreasing slagging tendency with increasing CS proportion, suggesting that the addition of CS mitigates the slagging risk during SS combustion. This mitigating effect arises because CS increases the Si and Al contents in the fuel while diluting the concentrations of basic oxides.

#### 3.2.3. Microscopic Morphology

**Figure 7:**
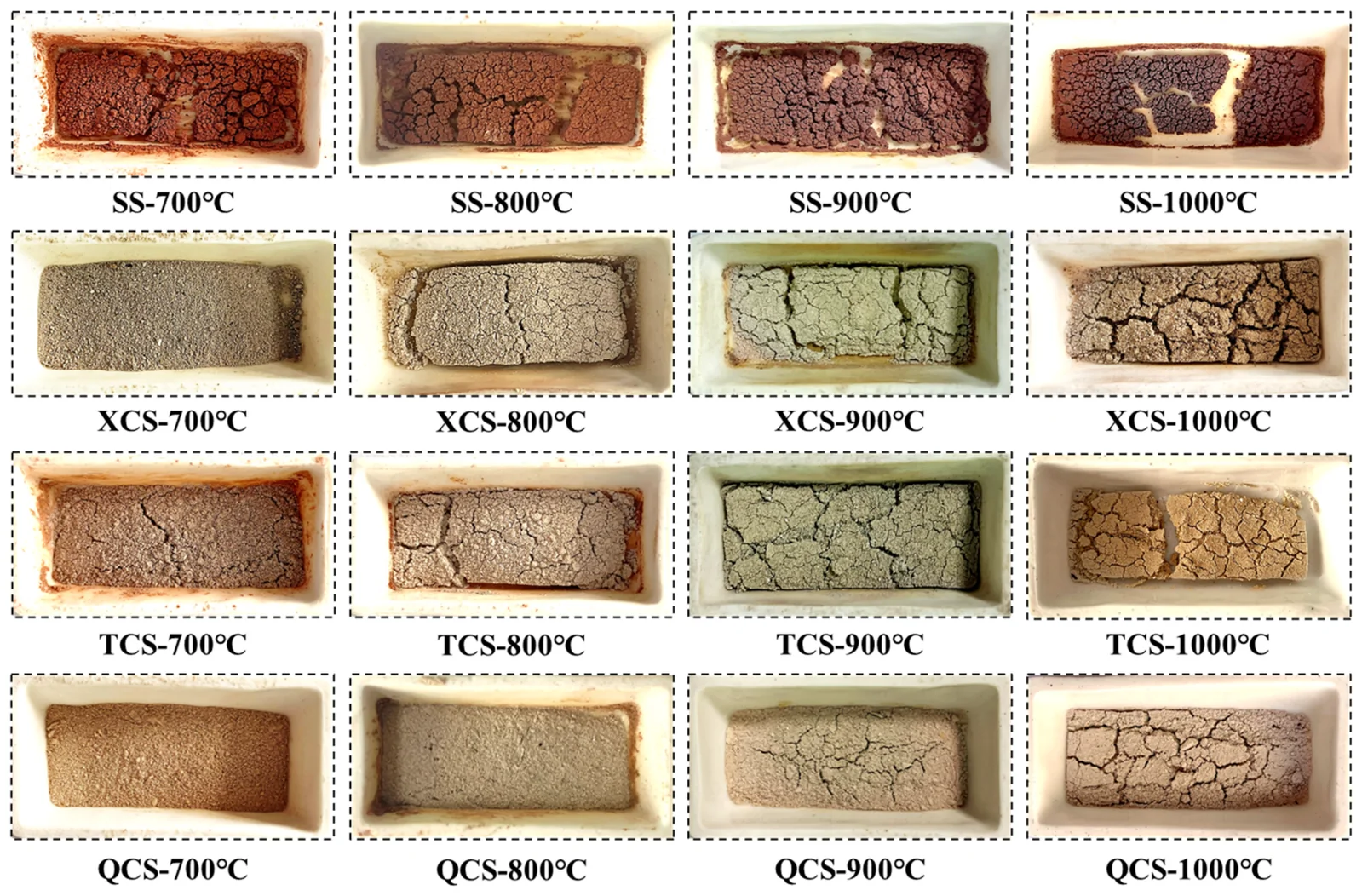
Macroscopic morphology of SS/CS at different temperatures.

To evaluate the applicability of the various evaluation indices for SS-CS combustion, a muffle furnace was used to prepare ash samples at a series of combustion temperatures. Afterwards, the macro- and micro-morphologies were analyzed. The macroscopic morphologies of the combustion products from SS, XCS, TCS, and QCS at 700–1000 °C (in increments of 100 °C) are shown in Figure 7.

It is evident that the apparent morphologies of the combustion products of SS and CS differed markedly at different temperatures. The ash generated from SS combustion was predominantly reddish-brown owing to its high Fe_2_O_3_ content, whereas the ashes from the three CS samples were grayish-white.

At 700 °C, the SS ash exhibited slight volumetric shrinkage; however, the particles remained loosely dispersed, with no evident melting or slagging phenomena observed. The three CS samples showed no volumetric shrinkage at 700 °C, and their ashes remained fluffy. At 800 °C, the SS ash exhibited more pronounced volumetric shrinkage and developed distinct surface cracks. The XCS and TCS ashes began to show volumetric shrinkage and fine cracks, yet still maintained a fluffy structure, whereas the QCS ash displayed no noticeable changes. As the combustion temperature increased further to 900 °C, the SS ash darkened in color and partially adhered to the porcelain boat, making it difficult to remove, and the XCS and TCS ashes exhibited further volumetric shrinkage, and surface cracks began to appear on the QCS ash. At 1000 °C, the SS ash exhibited pronounced agglomeration between particles, resulting in a hardened texture, and the TCS ash underwent further volumetric shrinkage, whereas the XCS and QCS ashes remained fluffy and soft.

**Figure 8:**
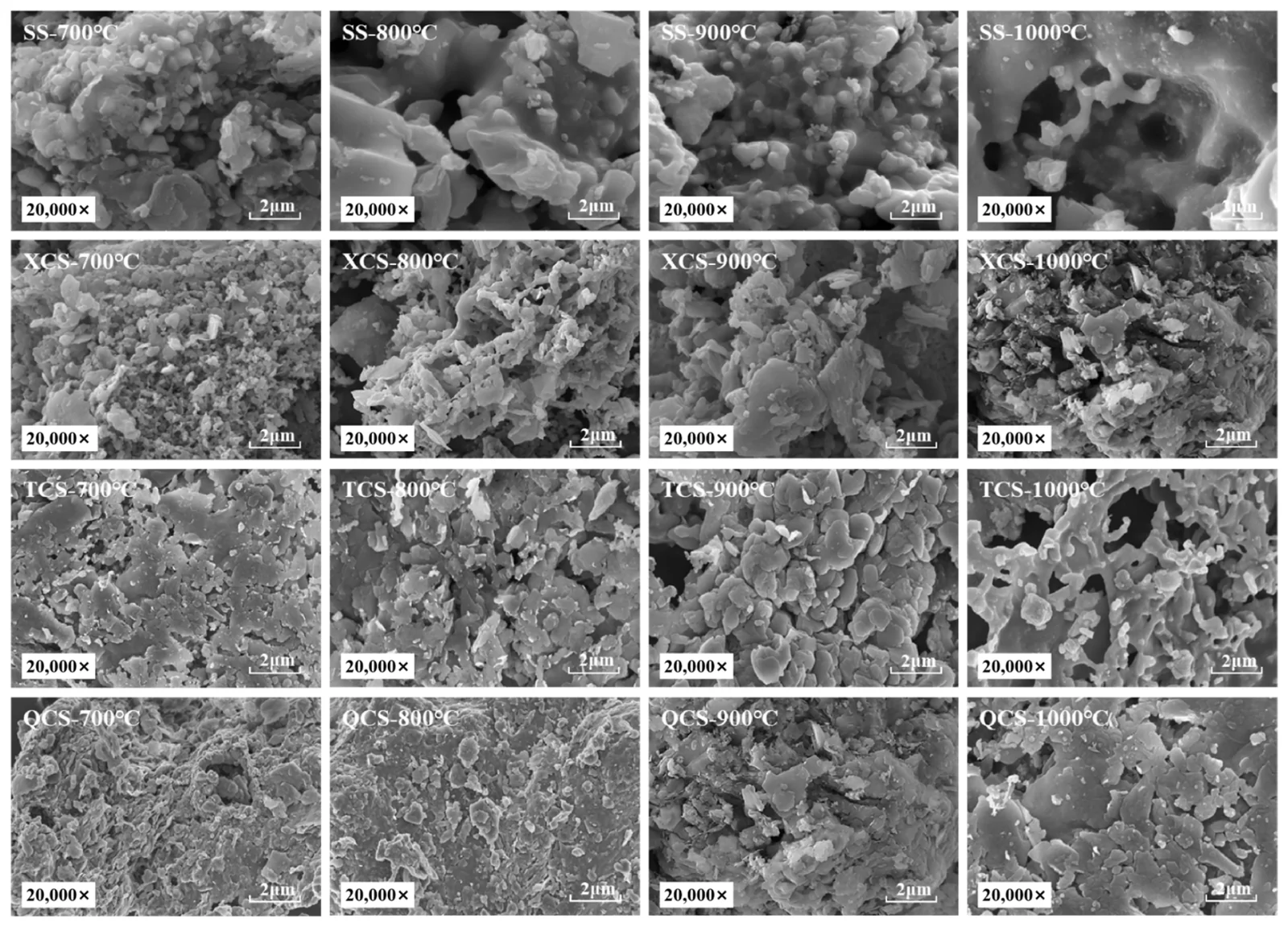
SEM images of SS/CS at different temperatures.

The microscopic morphologies of the aforementioned ash samples were further examined by SEM, as shown in Figure 8. At 700 °C, the SS ash comprised stacked blocky or flake-like particles, exhibiting a relatively dense surface with few pore structures, while no evident sintering was observed. As the combustion temperature increased to 800–900 °C, the ash surface became progressively smoother and denser, with some small crystallites beginning to spheroidize and adhere to the surfaces of larger crystallites. At 1000 °C, the ash underwent extensive sintering, during which the mineral crystallites melted into spherical droplets, coalesced with each other, and produced localized pore structures. The three CS ashes were primarily composed of flocculent or blocky particles stacked together, with XCS and QCS showing no evident melting or sintering throughout the combustion temperature range, whereas TCS exhibited partial melting and inter-particle adhesion at 1000 °C.

**Figure 9:**
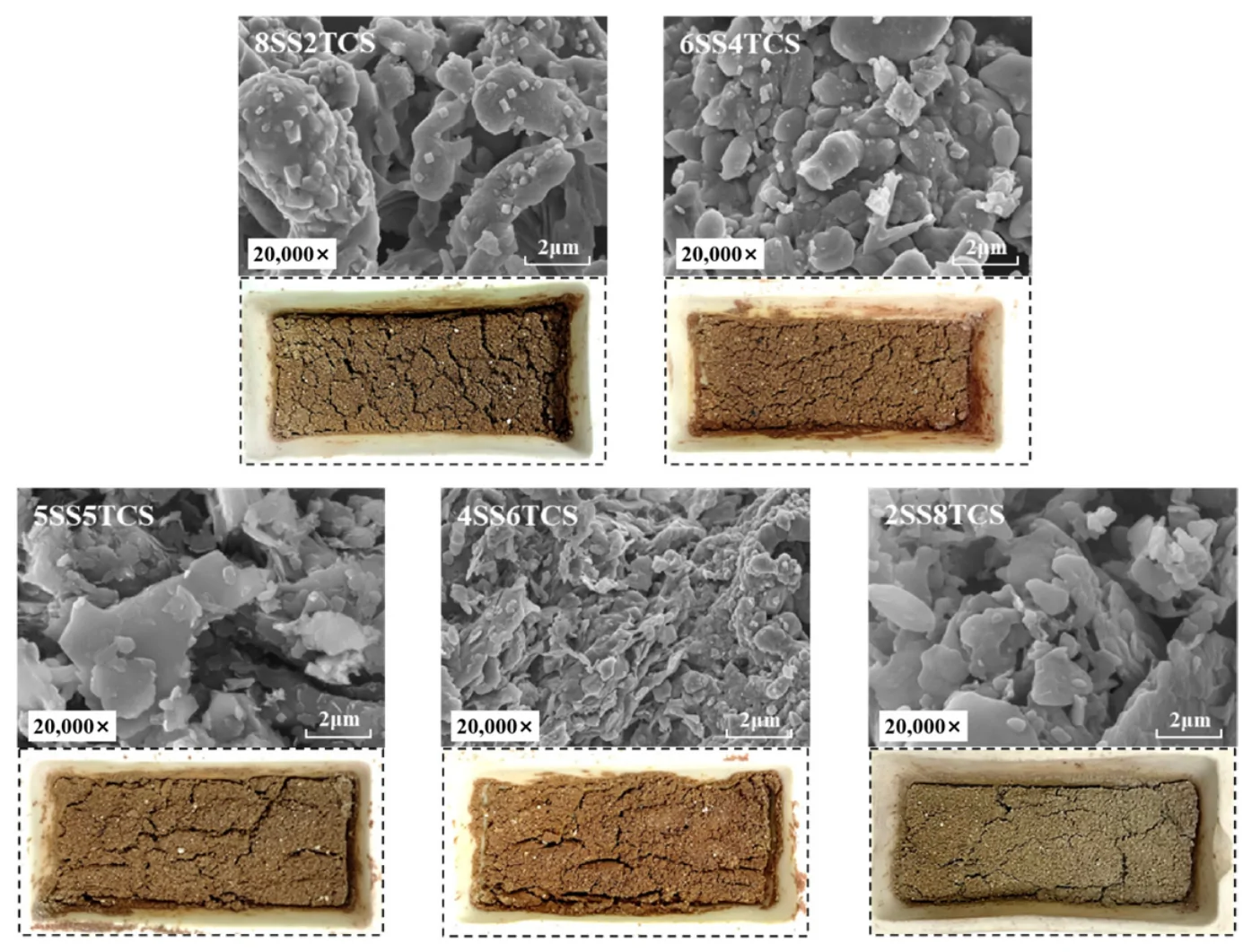
Macroscopic and microscopic morphology of SS-TCS at different temperatures.

Using SS-TCS as a representative case, the effect of the blending ratio on slagging characteristics was examined, and Figure 9 presents SEM images and photographs of the ashes from SS-TCS mixtures with different blending ratios after combustion at 900 °C.

When the TCS proportion was 20%, the slagging behavior of the blend was markedly improved compared with SS combustion alone. The 8SS2CS ash exhibited no evident agglomeration or sintering, showing only fine surface cracks. As the TCS proportion increased, the blend ash became progressively more fluffy, and its color gradually lightened. When the TCS proportion was increased to 80%, the macroscopic morphology of the blend ash closely resembled that of pure TCS. According to the SEM results, at 900 °C, the 8SS2CS ash exhibited melting and inter-particle adhesion. Localized spheroidization was observed on the surface of the 6SS4CS ash, whereas for the other three blending ratios, the ash particles remained loosely distributed with no evident melting under the same conditions.

In summary, the distinct slagging tendencies of SS and CS can be attributed to their differing inorganic compositions. SS exhibited a significantly higher slagging propensity than CS, in agreement with the predictions from all indices except *Fe*/*Ca*, confirming the applicability of these indices (excluding *Fe*/*Ca*) for assessing the slagging behavior of SS. The slagging tendencies of the three CS samples showed slight variations due to differences in composition, following the order TCS > XCS > QCS. This trend was consistent with the predictions of *R_s_* and *R_z_*, indicating that, in addition to acid-base oxides, the influences of sulfur content and other factors should also be fully considered when evaluating the slagging propensity of CS. For the blends, the addition of CS markedly reduced the fuel’s slagging propensity, with the extent of mitigation positively correlated with the CS proportion. This trend was consistent with the predictions from all the aforementioned indices except *Fe*/*Ca*. Although the addition of CS markedly mitigated the slagging propensity of SS, the blended fuels still exhibited a moderate to severe slagging tendency at most mixing ratios. Therefore, SS-CS co-combustion should be carried out with an appropriate blending ratio and at relatively low temperatures. The results indicate that when the CS proportion exceeds 40% and the combustion temperature is maintained below 900 °C, slagging can be effectively avoided.

## 4. Conclusions

This study systematically investigated the influence of CS diversity on the co-combustion characteristics and kinetics of SS-CS blends and further conducted a multidimensional analysis of ash slagging behavior during co-combustion to identify the most suitable indices for predicting the slagging propensity of the blended ash. The main conclusions are as follows:

(1) A multi-step kinetic process was observed in the combustion of both SS and CS. For SS, the activation energy showed an “N-shaped” distribution with increasing conversion, whereas that of CS decreased progressively as the reaction proceeded. In addition, the average activation energy of CS increased with its ash content.
(2) Introducing CS into the co-combustion process led to a decrease in the apparent activation energy. Specifically, with a TCS blending ratio of 50%, the activation energy was 20.0 kJ/mol less than that for SS burned individually. Correspondingly, the kinetic mechanism for fixed carbon combustion shifted from *f*(*α*) = (1 − *α*) to *f*(*α*) = (1 − *α*)^1.3^.
(3) SS exhibited a significantly higher slagging propensity than CS, with the three CS samples following the order TCS > XCS > QCS. Among the evaluation indices, *Fe*/*Ca* failed to accurately predict the extent of slagging for both SS and CS, whereas *R_s_* and *R_z_* showed good applicability for evaluating the slagging propensity of CS.
(4) CS addition brought about a clear improvement in the fuel’s slagging performance throughout the co-combustion of SS and CS. However, an appropriate blending ratio and a relatively low combustion temperature were still required. The laboratory results suggest that, for the SS–CS system investigated, a CS proportion above 40% and a combustion temperature below 900 °C are favorable for mitigating slagging.

Overall, the co-combustion behavior of SS-CS blends was significantly influenced by the type of CS, primarily due to differences in fixed carbon and ash content among the different CS samples. In particular, the slagging behavior was closely governed by both the amount and composition of the ash. Therefore, future research should further investigate the effects of individual ash constituents in CS on slagging behavior to optimize the industrial application of SS-CS co-combustion.

## Figures and Tables

**Table 1:** Proximate and ultimate analysis for SS and CS.

Samples	Proximate Analysis (Air-Dried Basis, %)				Ultimate Analysis (Dry Basis, %)					H/C	O/C	Q_ad.net_ (MJ/kg)
	M_ad_	V_ad_	A_ad_	FC_ad_	C_d_	H_d_	O_d_	N_d_	S_d_			
SS	2.44	47.44	46.18	3.94	26.18	3.94	17.09	4.22	1.24	0.15	0.65	10.84
XCS	0.21	14.31	17.87	67.61	72.27	3.35	3.41	1.19	1.87	0.04	0.05	26.15
TCS	0.38	13.04	51.00	35.58	37.70	2.17	2.33	1.03	5.57	0.06	0.06	15.19
QCS	0.26	9.66	69.58	20.51	24.05	2.12	2.98	0.38	0.71	0.09	0.12	8.18

Note: M, V, A, and FC denote moisture, volatile matter, ash, and fixed carbon, respectively. The subscripts ad and d denote the air-dried and dry bases, respectively.

**Table 2:** Inorganic component content in SS and CS.

Samples	Component Content (%)									
	SiO_2_	Al_2_O_3_	Fe_2_O_3_	SO_3_	CaO	K_2_O	ClO_2_	P_2_O_5_	MgO	ZnO
SS	13.5	4.6	43.6	4.5	10.6	4.6	1.0	10.4	2.7	1.7
XCS	42.0	24.6	9.0	14.0	3.1	1.4	2.0	0.4	-	0.1
TCS	44.9	27.4	12.1	7.9	2.7	1.4	0.8	0.5	-	0.1
QCS	59.1	29.5	4.1	0.5	0.9	2.6	-	0.1	-	-

**Table 3:** Common reaction mechanism models.

**Model**	Code	Mechanism	*f*(*α*)	*G*(*α*)
Reaction order model	F1	First-order	1 - α	$-\ln\left(1\;-\;\alpha \right)$
	F2	Second-order	${\left(1-\;\alpha \right)}^{2}$	${\left(1\;-\;\alpha \right)}^{-1}-1$
	F3	Third-order	${\left(1\;-\;\alpha \right)}^{3}$	$1/2\left[{\left(1\;-\;\alpha \right)}^{-2}\;-\;1\right]$
	F4	Fourth-order	${\left(1\;-\alpha \right)}^{3}$	$1/3\left[{\left(1\;-\;\alpha \right)}^{-3}\;-\;1\right]$
Geometrical contraction model	R1	One-dimensional	1	α
	R2	Two-dimensional	${2\left(1\;-\;\alpha \right)}^{1/2}$	${1\;-\;\left(1\;-\;\alpha \right)}^{1/2}$
	R3	Three-dimensional	${3\left(1\;-\;\alpha \right)}^{2/3}$	${1\;-\;\left(1\;-\;\alpha \right)}^{1/3}$
Random nucleation and growth model	A2	Two-dimensional	${2\left(1\;-\;\alpha \right)\left[-\ln\left(1\;-\alpha \right)\right]}^{1/2}$	${\left[-\ln\left(1\;-\;\alpha \right)\right]}^{1/2}$
	A3	Three-dimensional	${3\left(1\;-\alpha \right)\left[-\ln\left(1\;-\;\alpha \right)\right]}^{2/3}$	${\left[-\ln\left(1\;-\;\alpha \right)\right]}^{1/3}$
	A4	Four-dimensional	${4\left(1\;-\;\alpha \right)\left[-\ln\left(1\;-\;\alpha \right)\right]}^{3/4}$	${\left[-\ln\left(1\;-\;\alpha \right)\right]}^{1/4}$
Power law model	P2	Second power	$2\alpha^{1/2}$	$\alpha^{1/2}$
	P3	Third power	$3\alpha^{2/3}$	$\alpha^{1/3}$
	P4	Fourth power	${4\alpha }^{3/4}$	$\alpha^{1/4}$
Diffusion model	D1	One-dimensional	0.5α	$\alpha^{2}$
	D2	Two-dimensional	${\left[-\ln\left(1\;-\;\alpha \right)\right]}^{-1}$	α +(1 - α)ln(1 - α)

**Table 4:** Pre-exponential factor fitting results of SS/CS.

Samples	*A* (min^−1^)		*R* ^2^
	Stage 1	Stage 2	
SS	3.67 × 10^24^	4.86 × 10^12^	0.9878
XCS	9.31 × 10^15^	1.31 × 10^9^	0.9945
TCS	9.89 × 10^19^	1.96 × 10^9^	0.9884
QCS	5.43 × 10^13^	1.12 × 10^10^	0.9757

**Table 5:** Combustion kinetic parameters of SS-TCS.

Samples	*E_a_* (kJ/mol)	*f*(*α*)	*A* (min^−1^)
8SS2TCS	168.5	*f*(*α*) = (1 − *α*)^1.4^	3.91 × 10^10^
5SS5TCS	144.4	*f*(*α*) = (1 − *α*)^1.3^	6.94 × 10^10^
2SS8TCS	148.1	*f*(*α*) = (1 − *α*)	1.01 × 10^9^

## Data Availability

The original contributions presented in this study are included in the article. Further inquiries can be directed to the corresponding author.

## Appendix A

**Table A1:** Slagging tendency discriminant index.

Index Category	Discriminant Index	Test or Calculation Method	Criterion	Slagging Tendency
Ash fusion temperature (°C)	Deformation temperature (*DT*)	Ash fusion temperature tester	>1289	Low
			1108~1288	Medium
			<1107	Severe
	Softening temperature (*ST*)	Ash fusion temperature tester	>1390	Low
			1260~1390	Medium
			<1260	Severe
Ash composition	Base-to-acid ratio (*B/A*)	(Fe_2_O_3_ + CaO + MgO + Na_2_O + K_2_O)/(SiO_2_ + Al_2_O_3_ + TiO_2_)	<0.206	Low
			0.206~0.400	Medium
			>0.400	High
	Silica–alumina ratio (*Si/Al*)	SiO_2_/Al_2_O_3_	<1.87	Slight
			1.87~2.65	Medium
			>2.65	Severe
	Silica ratio (*G*)	(SiO_2_ × 100)/(Fe_2_O_3_ + CaO + MgO + SiO_2_)	>72	Slight
			65~72	Medium
			<65	Severe
	Iron–calcium ratio (*Fe/Ca*)	Fe_2_O_3_/CaO	<0.3	Non-slagging
			0.3~3.0	Severe slagging
			>3.0	Non-slagging
	Sulfur slagging index (*R_s_*)	*B*/*A* × *S_t,d_*	<0.6	Slight
			0.6~2.0	Medium
			2.0~2.6	Strong
			>2.6	Severe
	Comprehensive ash index (*R_z_*)	1.24*B/A* + 0.28*Si/Al* − 0.0023*ST* − 0.0019*G* + 5.4	<1.5	Low
			1.5~2.5	Medium
			>2.5	High
